# Some Scientific Aspects of Insanity

**Published:** 1891-11-21

**Authors:** 


### Some Scientific Aspects of Insanity.

### III.—NERVE TISSUE.*

The human brain contains in comparison much more gray matter than the brain of any other animal, and it is for this reason that the surface of the human brain is so crumpled and convoluted. On looking at the brain of one of the lower mammals, such as a cat or a dog, we find that the surface is comparatively smooth, and does not exhibit so many ridges and depressions. The surface of the human brain is so crumpled and convoluted for the same reason that you crush up your pocket handkerchief when you place it your pocket, to make the best use of the available space.

To return now to the simile of the shell fish. From each of the larger cells in the gray matter a slender nerve fibre passes into the white matter of the brain. As the cells are very numerous these fibres are also numerous, and they converge towards the base of the brain in order to enter into the spinal cord. These are what may be termed the claws of the nervous system. We shall hereafter call them the motor or outgoing fibres. An equal number of nerve fibres run up from the skin and organs of sense through the spinal cord to the gray matter of the brain, these correspond to the feelers of the shell fish. We shall hereafter call them the sensory or in-going fibres. Besides the out-going and in-going fibres there are nerve fibres connecting one part of the gray matter with another part, and one part of the hemisphere with a corresponding part on the other side. The white matter of the brain then is entirely composed of nerve fibrils of three kinds, first, out-going fibres; second, in-going fibres: third, connecting fibres.

We shall next consider the spinal cord. Whereas in the brain the gray matter envelopes the white matter, in the spinal cord, the order is reversed and the gray matter occupies the centre of the cord and the fibres of the white matter surround it. The spinal cord springs from the base of the brain, its junction with the latter organ being marked by an enlargement of the cord termed the medulla oblongata. The cord is securely protected within the backbone, the processes of the segments of which surround it very much in the same way as a number of rings superimposed one upon another would enclose a soft structure in the interior of the tube thus formed. Like the brain, the cord is divided throughout its whole length by two grooves, one in front and one behind, into a right half and a left half. As I have already pointed out, the gray matter is in the centre expanding like a fan into each half of the cord and narrowing at the junction between the two halves. In the centre of this narrow connecting bridge is situated a very fine tube or central canal which runs from about the centre of the brain to the lower end of the spinal column. It is around this central canal that the gray matter of the cord and central part of the brain is collected, and it is from this gray matter that nearly all the nerves of the body spring. I have spoken about the outgoing and the ingoing fibres of the brain as being the claws and the feelers of the nervous system; so they are, it is true, but both the outgoing and the ingoing brain fibres end in some part of this long tract of gray matter which I have just described. That is to say, they do not pass continuously to and from every part of the body, but are interrupted, just as a telegraph wire does not pass straight from London to a small provincial village, but is interrupted at the office in a large town which the village adjoins. Take, for example, a sensation conveyed from the eye through the optic nerve to the brain. The optic nerve does not pass straight into the gray matter of the brain, but is interrupted first in the gray matter surrounding the top of this canal. On the other hand, take an out-going message conveyed from the brain which conveys an order, by means of which anything is seized with the hand. The fibre conducting the message does not pass directly down the arm to the muscles of the hand, but is interrupted in the gray matter of the cord at a point, roughly speaking, situated between the shoulders. From this point new nerve fibres arise from the cells in the grey matter of the cord, and pass to the muscles of the hand. The white matter of the cord is like the white matter of the brain, composed of descending or out-going fibres, of ascending or in-going fibres, and of fibres connecting one part of the cord with another.

From the upper part of the tract of gray matter situated within the brain cavity there arise twelve pairs of nerves; four of these pairs are the nerves of the special senses of sight, smell, taste, and hearing, which convey impressions from each of these organs to the brain. The remaining eight are concerned in the movements of the eye and face and the regulation of such important functions as the action of the heart and the breathing. From the spinal cord thirty-one pairs of nerves arise, each pair of nerves springing by tw

* This article should have preceded the last one, but owing to a clerical error in numbering, the second and third sections of the series have been transposed. It is hoped that not much inconvenience will result from the temporary break in the continuity of the subject.—Author.

roots at the same level from opposite sides of the cord. These roots are arranged in the following manner. One root springs from the front the other from behind at the same level. On the posterior root is a small bead-like enlargement which always distinguishes it. The anterior root is plain. The roots join beyond the spinal cord to form one large nerve trunk.

NERVE TRUNKS.

The nerve trunk is composed of out-going fibres, and in-going fibres. The out-going fibres conveying motor impressions from the central nervous system to the various parts of the body; the in-going fibres conveying sensory impressions to the nervous system. In entering the spinal cord the in-going nerves pass through the posterior root and immediately cross over to the opposite side of the cord through which they ascend without interruption to the brain.

A nerve trunk or common nerve of the body is surrounded by a sheath which binds its fibres firmly together. The fibres within the sheath are further sub-divided into small bundles, each with its own sheath. In its course through the body it divides and sub-divides exactly as a tree branches and each little branch carries with it a portion of the sheath. Ultimately, the branches become so fine owing to subdivision that they can scarcely be seen without microscopic aid. Some of them end in the skin, forming the commencement of the in-going nerves, others end in the muscles where each little twig is applied to each muscular fibre by means of a minute plate-like arrangement. These, of course, form the terminations of the motor or out-going nerves.

